# The COVID-19 pandemic masks the way people perceive faces

**DOI:** 10.1038/s41598-020-78986-9

**Published:** 2020-12-21

**Authors:** Erez Freud, Andreja Stajduhar, R. Shayna Rosenbaum, Galia Avidan, Tzvi Ganel

**Affiliations:** 1 Department of Psychology and the Centre for Vision Research, York University, Toronto, Canada; 2 Rotman Research Institute, Baycrest Health Sciences, Toronto, Canada; 3 Department of Psychology, Ben-Gurion University of the Negev, 8410501 Beer-Sheva, Israel; 4 Department of Cognitive and Brain Sciences, Ben-Gurion University of the Negev, 8410501 Beer-Sheva, Israel

**Keywords:** Human behaviour, Perception

## Abstract

The unprecedented efforts to minimize the effects of the COVID-19 pandemic introduce a new arena for human face recognition in which faces are partially occluded with masks. Here, we tested the extent to which face masks change the way faces are perceived. To this end, we evaluated face processing abilities for masked and unmasked faces in a large online sample of adult observers (n = 496) using an adapted version of the Cambridge Face Memory Test, a validated measure of face perception abilities in humans. As expected, a substantial decrease in performance was found for masked faces. Importantly, the inclusion of masks also led to a qualitative change in the way masked faces are perceived. In particular, holistic processing, the hallmark of face perception, was disrupted for faces with masks, as suggested by a reduced inversion effect. Similar changes were found whether masks were included during the study or the test phases of the experiment. Together, we provide novel evidence for quantitative and qualitative alterations in the processing of masked faces that could have significant effects on daily activities and social interactions.

## Introduction

Faces are among the most informative and significant visual stimuli in human perception. Brief presentation of a person’s face readily exposes their identity, gender, emotion, age, and race^1^. The unprecedented efforts to minimize the effects of the novel coronavirus include a recommendation (and in most countries, a requirement) to wear face masks in public to reduce virus transmission^2,3^. Around the globe, mask-wearing is being introduced as a new requirement as governments ease restrictions to reopen the economy. This new constraint introduces a whole new arena for face recognition in which typical and commonly encountered faces are partially occluded. Given the importance of intact face processing to everyday life and to social interactions, it is imperative to characterize how wearing masks might hamper these abilities.

A reduction in recognition of masked faces is predicted by previous research. In one such study, a small sample of human participants were found to be impaired in recognition of masked faces^4^. In a separate study, recognition accuracy was similarly reduced when different features (eyes, mouth, nose) of the face were systemically removed^5^. Other studies showed that the recognition of facial expressions is modulated if the lower part of the face is occluded by a scarf or ethnic related headdresses^6^. More recently, it was found that surgical masks impair face perception of familiar and unfamiliar faces^7^. These findings are also consistent with experiments in which Gaussian masks (i.e., bubbles) were parametrically added to face images, revealing that the mouth and eye regions are among the most important sources of information that support face identification^8^. Together, these previous studies suggest that the occlusion of specific face features can impair face recognition abilities. However, the extent of this impairment and the perceptual-cognitive mechanism that mediates it remain unclear. The current study aims to address these outstanding questions.

Normal face perception is characterized by a unique processing style that emphasizes holistic aspects of the face rather than its specific features^9,10^. Maurer and colleagues^10^ distinguish between three types of configural processes that contribute to face perception: 1. detecting the first-order relations that define faces (i.e., two eyes above a nose and mouth), 2. integrating the features into a coherent gestalt, and 3. processing second-order relations between features. Face masks conceal the lower part of the face, including the mouth and nose area. This information is critical for processing the face as a whole, and, therefore, it is expected that at least some aspects of holistic processing would be disrupted by a mask covering part of a face.

Previous research demonstrated a relationship between holistic processing and face perception performance. For example, face recognition accuracy was found to be correlated with the extent to which observers process the faces holistically^11^. Neuropsychological investigations have shown that alterations of holistic processing are associated with deficits in face perception abilities. For example, disruption in holistic processing has been observed in individuals with acquired^12–14^ and congenital^15,16^ prosopagnosia, further emphasizing the significance of this form of processing to face perception. The importance of holistic processing has also been demonstrated in observers with normal face perception abilities. Indeed, previous research documented a robust deficit in the perception of inverted vs. upright faces^17^ that is termed the face inversion effect (FIE). The FIE is disproportionately larger for faces compared with other visual categories and is often considered as a signature of disrupted holistic processing of faces^17–19^. Notably, it was demonstrated that the lower part of the face (mouth region) has a disproportional contribution to holistic processing compared with the upper part (eye region), such that the inversion manipulation impairs recognition of the mouth much more than the eyes^20^.

The COVID-19 outbreak has led to widespread adoption of face masks as a primary line of protection. Given that previous research has already shown that occlusion of face parts hinders face perception abilities^4^ and can also reduce holistic processing^21^, we predict that face masks will disrupt face perception abilities and change the way faces are processed. To directly test these predictions, we used the Cambridge Face Memory Test (CFMT)^22,23^, which is a widely used and established test of face recognition abilities. Specifically, we created an identical version of this task in which faces were masked, and examined performance differences across these two versions. To test whether any reduction in face perception is accompanied by a qualitative change in holistic face perception, we also constructed upright and inverted versions of the test phase. The study was conducted online in a large sample (nearly 500 participants across two experiments) and provides a comprehensive, novel account of face recognition abilities with and without masks.

## Methods

### Participants

Two hundred and ninety-three participants (151 females) with a mean age of 25.5 years (SD = 7.4, range 18–57) were recruited online (https://www.prolific.co/) during the period of May–June 2020 (2–3 months after mask wearing became common). Participants were compensated for their time (~ $6 CAD for 30 min). This sample size was chosen to ensure robust statistical power and is consistent with previous large-scale studies^24^. All experiments were performed according to relevant guidelines and regulations of the ethics review board at York University. All participants provided informed consent. Data and analysis code for all experiments are available on the Open Source Framework (osf.io/yj38h) under CC-By Attribution 4.0 International license.

### Materials

The extended version of the CFMT^22,23^ was used to assess face perception abilities. The standard CFMT includes three phases (total of 72 trials) with an increasing level of difficulty. The first phase (easy) involves learning to recognize six unfamiliar male faces from three different viewpoints and then testing recognition of these faces in a three-alternative forced-choice task. The second phase involves a refresher in which the six faces are presented simultaneously from one (frontal) viewpoint followed by testing from novel viewpoints and lighting conditions. The third (difficult) phase is similar to the second phase but includes test images with added visual noise. The long form of the CFMT includes additional 30 trials with an even higher level of difficulty in which novel images varying in pose, emotional expression, and the amount of information available are presented. This latter part is typically used to identify super-recognizers (individuals with an extraordinarily high face recognition memory)^23^, while the first two phases are more sensitive to detecting basic performance and potential deficits within face perception abilities^25,26^. For the current study, we limited most analyses to the standard form of the CFMT (level 1–3), but also include an analysis of the entire CFMT test divided into different levels of difficulty.

### Procedure

**Figure 1:**
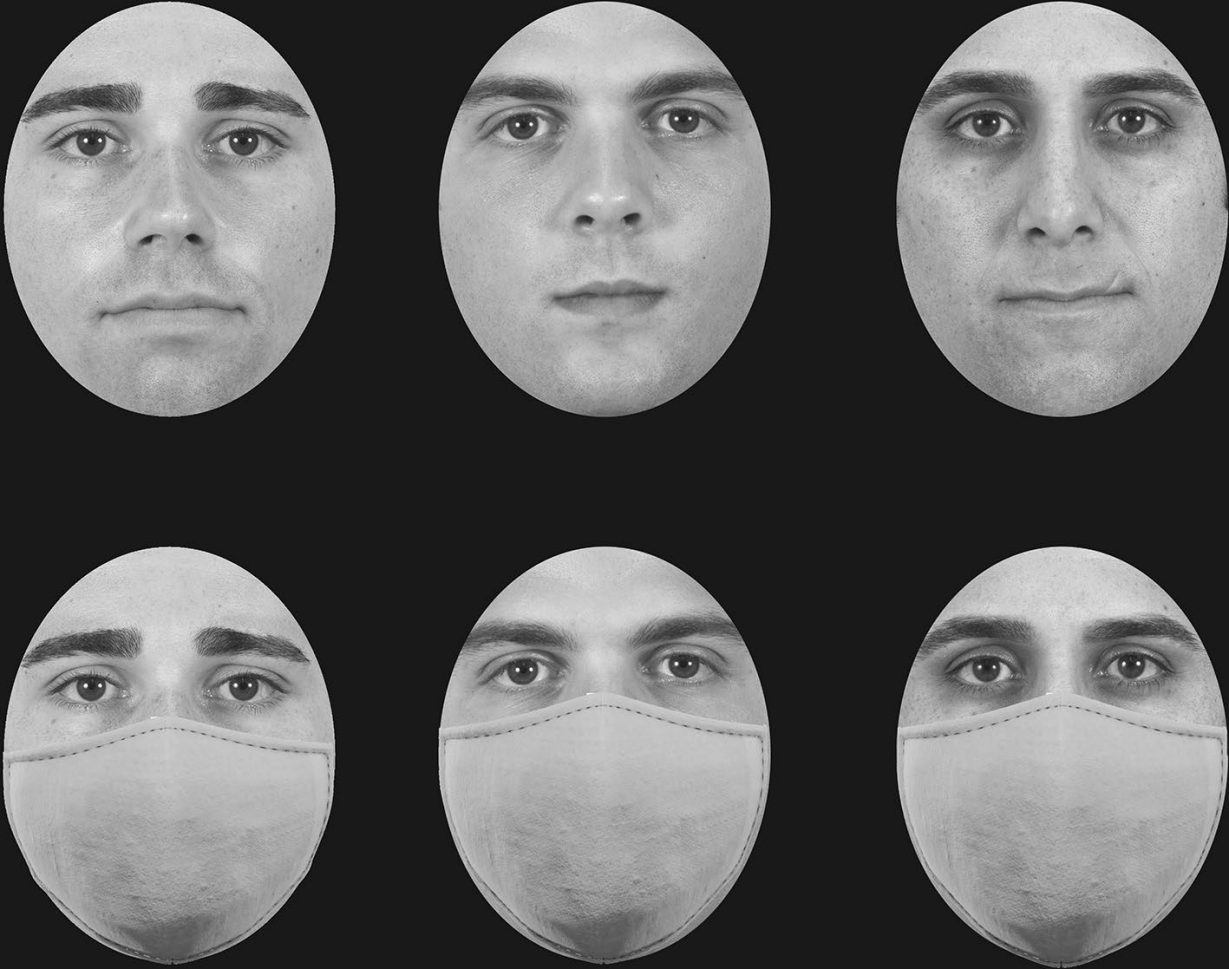
Examples of faces with and without masks similar to the ones used in the experiment. Faces are reproduced with premission from the Chicago Face Database^44^.

Participants were randomly assigned to one of two groups. The first group completed the original CFMT (faces without masks), while the second group completed a modified version of the CFMT in which an identical face mask was added to all faces (Fig. 1). To explore the processing style of faces with and without masks, each participant completed the test twice, once with upright faces and once with inverted faces^22^. Block order (upright or inverted) was counterbalanced between participants. Accuracy scores (0–72) for upright and inverted faces were computed and served as the dependent variable. Statistical analyses were conducted using in-house code written in Python and JASP^27^.

## Results

We explored the extent to which face masks led to reduction in face recognition abilities. To this end, participants completed the CFMT with upright and inverted faces (a manipulated within-subjects design) while the faces were either masked or non-masked (manipulated between-subjects). We also included gender as a between-subject variable, as previous research documented an advantage in face recognition abilities in female participants^24^.

**Figure 2:**
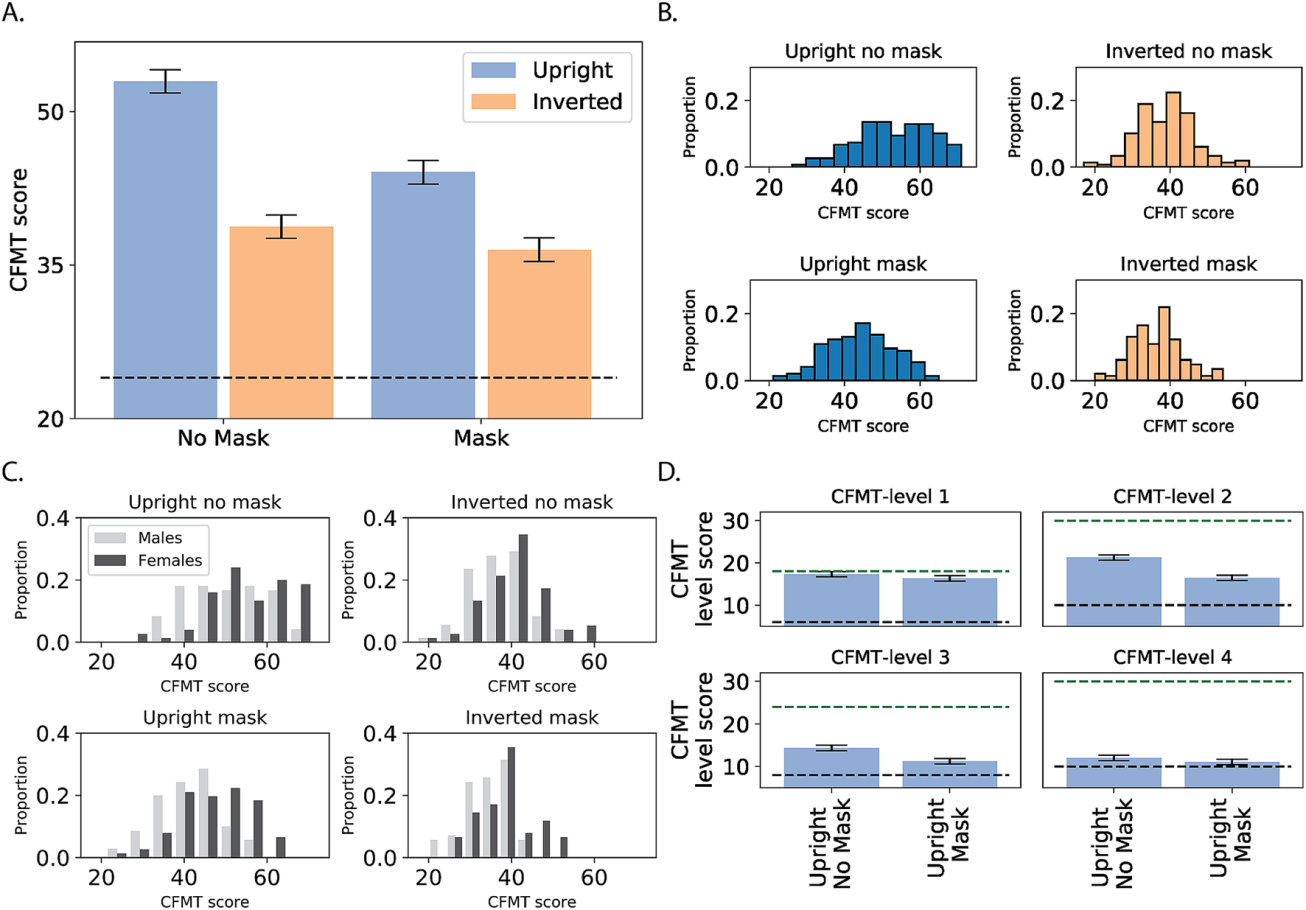
Results of Experiment 1: (**A**) Results of the CFMT experiment for non-masked and masked and faces. The dashed black horizontal line represents chance level (also in panel **D**). Performance was significantly impaired for masked faces. An inversion effect was found for masked and for non-masked faces, but it was significantly reduced for masked faces. For all figures, error bars represent the 95% confidence interval for the main effect of group (mask/no mask). (**B**) Distribution of CFMT results across the different conditions. (**C**) Distribution of the CFMT results across the different conditions separately for female and male participants. Females performed better than males across all conditions. (**D**) Analysis of the separate phases of the CFMT. An advantage for non-masked faces was found across all levels, including level 4, in which overall performance was close to chance level. The green dashed horizontal line represents the maximal score.

Figure 2A shows the group averages across conditions on the standard CFMT. The results show a robust alteration in face recognition abilities when masks are added to faces. In particular, there was a major decrease in performance (about 15%) for masked compared to non-masked faces.

A repeated-measures ANOVA with mask type (mask, no-mask) and orientation (upright, inverted) was conducted. We found a main effect of mask [F_(1,289)_ = 46.68, *p* < 0.001, η_p_^2^ = 0.13]. The mask effect was accompanied by a strong inversion effect [F_(1,289)_ = 629.5, *p* < 0.001, η_p_^2^ = 0.685] reflecting the well-documented advantage for upright faces. Importantly, these main effects were qualified by a two-way interaction between face orientation and group [F_(1,289)_ = 59.22, *p* < 0.001, η_p_^2^ = 0.17] (see Supplementary Materials for a full statistical report).

Planned comparisons showed that the inversion effect was evident for both non-masked [mean FIE: 14.2 points; F_(1,289)_ = 499, *p* < 0.001] and masked faces [mean FIE: 7.57 points; F_(1,289)_ = 163, *p* < 0.001], but it was significantly smaller for the latter, pointing to a qualitative difference in the processing style of masked faces. The inclusion of a mask led to a significant decrease in holistic processing and, in turn, to a more local, feature-based processing style. Importantly, this effect could not be attributed to a floor-performance effect, as performance for the inverted masked faces (36 ± 6) was well above chance level (24).

Consistent with previous research^24,28^, a main effect of gender was found in face recognition abilities, with females performing better than males [F_(1,289)_ = 32.51, *p* < 0.001, η_p_^2^ = 0.1]. This advantage was found across all mask conditions [F < 1, Fig. 2C].

Finally, as the CFMT is composed of phases encompassing different difficulty levels, we analyzed whether the effect of masks was mediated by difficulty level (Fig. 2D). Notably, for this analysis we also included the fourth, most difficult level that is typically used to identify individuals with superior face recognition abilities^23^. Because each level has a different number of items, we used percent accuracy (rather than absolute CFMT score) as the dependent variable for this analysis. The repeated-measures ANOVA showed a robust effect of group (mask vs. no-mask) [F_(1,291)_ = 32.51, *p* < 0.001, η_p_^2^ = 0.1] that was mitigated by a three-way interaction between group, orientation and difficulty level [F_(1,291)_ = 12.3, *p* < 0.001, η_p_^2^ = 0.04] (for the full statistical analysis, see Supplementary Materials). Notably, planned comparisons showed a performance advantage for non-masked faces across all levels of difficulty [Fs > 13, *p* < 0.001] excluding the last level [F_(1,291)_ = 2.05, *p* = 0.15]. The decrease in the effect at the most difficult level (level 4) might reflect a floor effect, as the average score (for masked and non-masked faces) approached chance level (33%). These results emphasize the finding that face masks impair face perception abilities across all levels of difficulty, even in the easiest condition in which only one facial identity is presented throughout the learning phase.

## Experiment 2

The results of Experiment 1 show a robust decrease in face recognition abilities for masked faces compared to non-masked faces. Additionally, we found evidence for a qualitative change in the way masked faces are processed, with reduced holistic processing of masked faces.

The CFMT task is composed of a learning phase and a test (recognition) phase. Notably, previous research, using various other paradigms, found that these two phases differ in their processing characteristics, for example, as evident in different eye movement patterns^29,30^. Hence, an outstanding question is whether the reduction in face recognition performance for masked faces could be attributed to the study phase, the test phase, or to both. To this end, in Experiment 2, participants completed the CFMT test but were now exposed to masked faces only at study or at test.

## Methods

### Participants

A new group of 203 participants (mask at study: 102 participants, mask at test: 101 participants; 103 females) with a mean age of 27 years (SD = 9.49, range 18–70) were recruited online (https://www.prolific.co/). Participants were compensated for their time (~ 6$ for 30 min).

### Materials and procedure

The materials and procedure were similar to those used in Experiment 1, except for the following changes. During the CFMT task, masked faces were included only during the study phase (mask-at-study) or alternatively during the test phase (mask-at-test). The “study phase” includes only the familiarization images (for each of the CFMT phases). Following completion of the CFMT experiment with upright and inverted faces (counterbalanced order), all participants completed the PI20, a 20-item questionnaire for assessing congenital prosopagnosia symptoms based on self-report^31^. Difficulties with face processing are scored on a Likert scale ranging between 1 and 5, with lower scores reflecting better perceived face recognition ability. We also added 3 new questions pertaining to the perception of masked faces (extended PI20). These items were: (1) “My face recognition ability is worse when people wear masks,” (2) “When people wear masks, I often mistake people I have met before for strangers,” and (3) “When people wear masks, without hearing their voices, I struggle to recognize them”.

## Results

**Figure 3:**
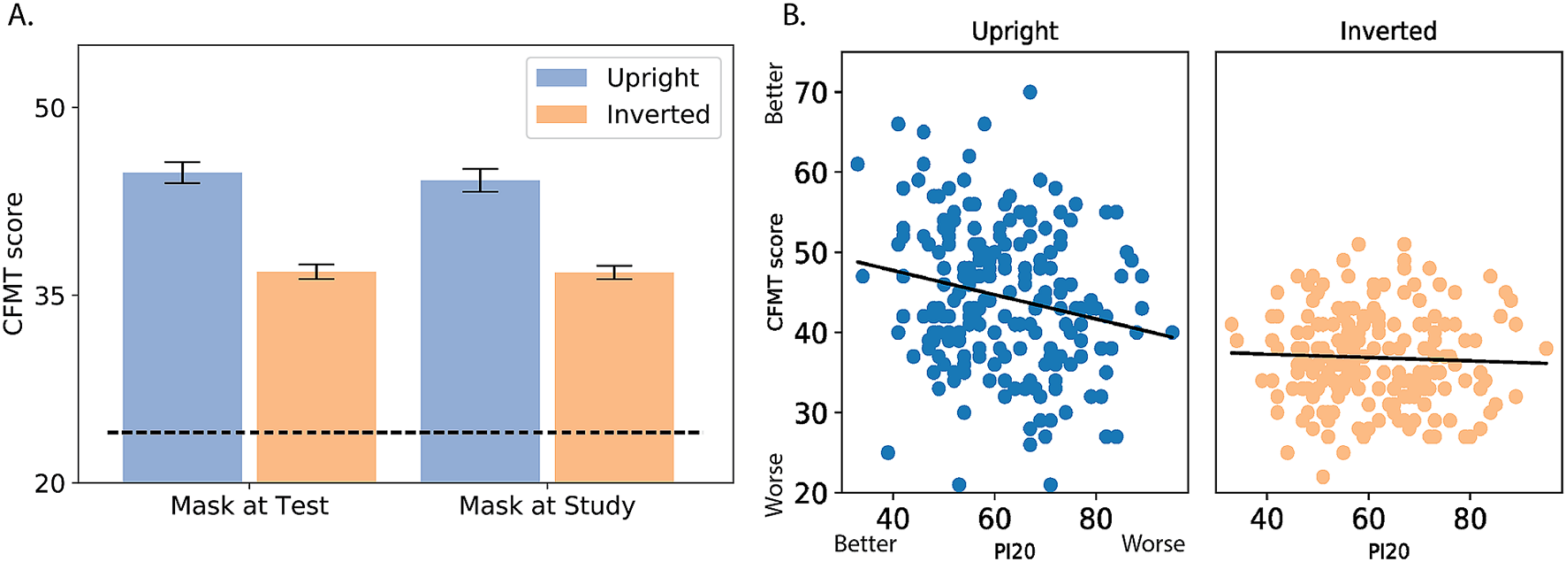
Results of Experiment 2: (**A**) Results of the CFMT experiment when faces were masked only during the test phase or the study phase. The results are similar to those obtained from the group in which faces were masked during both study and test phases. (**B**) Correlation between self-reported face perception abilities (extended PI20) and the CFMT results for upright and inverted faces.

To explore whether the effect of face masks on face recognition depends on the phase in which they are presented, participants completed the CFMT when faces were masked only during the study or the test phase. The results (Fig. 3A) were similar to those obtained in Experiment 1.

An ANOVA with group (mask, no-mask, mask at study, mask at test), gender, and face orientation revealed a robust inversion effect [F_(1,488)_ = 692, *p* < 0.001, η_p_^2^ = 0.58] as well as a main effect of group [F_(1,488)_ = 22.29, *p *< 0.001, η_p_^2^ = 0.12]. Critically, planned comparisons showed that mask covering during either the test phase [F_(1,488)_ = 33.57, *p* < 0.001] or study phase [F_(1,488)_ = 37.65, *p* < 0.001] led to reduced CFMT scores compared with the non-mask group. However, no differences were found between the other conditions. The inclusion of a face mask led to similar impaired performance regardless of whether the mask was included in the test or study phase [Fs < 1].

The two main effects were qualified by a two-way interaction [F_(1,488)_ = 25 , *p* < 0.001 , η_p_^2^ = 0.13], as the inversion effect was greater for the no-mask group compared with the other three groups [F_(1,488)_ = 75.41, *p* < 0.001]. The inversion effect was similar in magnitude for the three mask conditions [Fs < 1] (see Supplementary Materials for description of full statistical levels of inversion). These findings suggest that the inclusion of a mask hampers performance to a similar extent and leads to a similar decrease in holistic processing regardless of whether the mask is included at study, at test, or in both experimental phases. We note that in accordance with the results of Experiment 1, a main effect of gender was found [F_(1,488)_ = 31.6 , *p* < 0.001 , η_p_^2^ = 0.13], again pointing to superior face perception abilities among females.

Finally, we correlated the CFMT scores with self-reported face processing abilities as indicated by the extended 20-item Prosopagnosia index (PI20 + 3 additional mask-related questions; see “Methods”). Because we did not identify any differences between the two groups, we collapsed the correlational analysis across all participants (Fig. 3B). The results showed a weak correlation between the upright CFMT score and the extended PI20 score [r =  − 0.2, *p* < 0.005, CI − 0.33, − 0.07], (A similar result was obtained for the original PI-20 [upright: r =  − 0.21, *p* < .005, CI − 0.34, − 0.08; inverted: r =  − 0.04, *p* = .52, CI − 0.18, 0.09) such that better CFMT scores were associated with a lower score on the extended PI20 (i.e., better self-reported face perception abilities). A similar correlation was not found for inverted faces [r =  − 0.04, *p* = 0.52, CI  − 0.18, 0.09]. We note that previous studies reported higher correlations between face perception abilities and the PI20^31,32^ (e.g., r =  − 0.68 n = 110 data from ^32^; difference from the current reported correlation -Fisher r-to-z transformation—Z = 5.28, *p* < 0.001). However, the inclusion of the masked faces in the present study might have led to such reduced correlation.

## Discussion

Face masks are an important tool in the effort to minimize COVID-19 virus transmission and, as a result, are increasingly prominent in everyday social interactions. Here, we evaluated the extent to which face masks lead to a decrease in face perception abilities. We have documented quantitative and qualitative alterations in face processing abilities for masked faces. In Experiment 1, we found that the face masks led to a robust decrease in face processing abilities measured by the well-established CFMT. This quantitative reduction was accompanied by a weaker inversion effect, suggesting that the processing of masked faces is less holistic.

Experiment 2 extended these findings in showing that including a face mask in only one of the CFMT phases (i.e., either study or test) led to a similar deficit in face recognition abilities and to reduced holistic processing. This finding corroborates the results from Experiment 1 and further suggests that the face masks can alter different aspects of the face recognition system. Together, these findings imply that face processing abilities are highly susceptible to the inclusion of masks. Below we discuss plausible mechanisms that could account for the observed changes in masked face processing.

### Reduced inversion effect for masked faces

A key finding in the current experiment is the reduction of the face inversion effect for masked faces. In particular, for faces with no masks, we found a decrease of 14 points (26%) in the CFMT score for inverted faces, while a smaller inversion effect (7 points, 16%) was found for masked faces. The inversion of a face makes it difficult to extract configural relationships between face parts^17–19^, and, therefore, the twofold smaller inversion effect for masked faces can be taken as evidence that holistic processing is largely reduced (though not entirely abolished). Thus, the processing of masked faces relies more heavily on their available features rather than on configural or holistic information. Future research should utilize other measurements of holistic processing such as the Garner interreference task^33,34^ to further characterize the changes along the processing style of faces.

According to Rossion^35^, the inversion effect reflects a reduction in holistic processing and a sequential, spatially restricted processing of face features. This view can account for the current results. In particular, the upright masked faces are processed in a less holistic manner, resulting in reduced face perception abilities. For the inverted masked faces, feature processing is spatially limited, and, therefore, the effect of the mask is less evident, leading to a reduced face inversion effect. A similar alteration of face perception in general, and of holistic processing in particular, has been found within a different context—namely, the other race effect^36^. Michel et al.^37^ found a smaller face composite effect^38^ for other race faces compared with same race faces. This effect was interpreted as evidence for reduced holistic processing of other race faces. Together, these findings support the co-occurrence of a reduction in face perception abilities and a disruption of holistic face processing.

### Individual differences in face perception

Previous research already alluded to the remarkable individual differences in face perception abilities even within the normal population. At the extremes (or outside) of the normal range, there are individuals who are exceptionally good at face perception (i.e., super-recognizers)^23,24^ but also individuals with severe deficits in face perception (i.e., prosopagnosia)^39,40^.

Here we documented high variability in face perception abilities (see Figs. 2B, 3B) alongside a consistent advantage for females across all conditions and in both experiments, consistent with previous results^24,41^ (faces with and without masks; see Fig. 2C). We also replicated the relationship between self-reported face perception abilities and face perception performance. Notably, this effect was weaker than previously reported^31,32^, but this might be due to the use of masked faces to assess face perception abilities.

Recently it was reported that eye-movement patterns of faces also exhibit between-subject variability. In particular, four idiosyncratic but consistent, natural eye-pattern clusters were discovered. Those eye-movement patterns can be clustered based on the main focus of fixations on (1) the left eye, (2) the right eye, (3) the nasal bridge, and (4) the nose and upper lip. However, these patterns of fixation did not predict face perception abilities^42^. Given that masks occlude parts of the nose and the mouth, it is reasonable to assume that observers who exhibit preferential fixation to parts of the faces outside the region of the eyes in (3) and (4) would be more adversely affected by masks. Given that the current data were collected online, we could not evaluate this hypothesis. However, future research should examine this issue.

## Conclusion

The current study provides novel evidence for quantitative and qualitative changes in the processing of masked faces. These changes in performance, together with the alteration along the processing style of partially occluded faces, could have significant effects on activities of daily living, including social interactions, as well as other situations involving personal interactions such as education. Previous research already indicated that reduced face perception abilities following age-related macular degeneration is accompanied by negative consequences of social disengagement, a decrease in the level of social confidence, and a general decrease in quality of life^43^. Given that wearing masks has rapidly become an important norm in countries around the globe, future research should explore the social and psychological implications of this behavior.

## Supplementary Information

Supplementary Information.

## References

[1] Tsao DY, Livingstone MS (2008). Mechanisms of face perception. Annu. Rev. Neurosci..

[2] Canada, P. H. A. of. Coronavirus disease (COVID-19): Measures to reduce COVID-19 in your community. *aem* https://www.canada.ca/en/public-health/services/diseases/2019-novel-coronavirus-infection/prevention-risks/measures-reduce-community.html#w (2020).

[3] CDC. Coronavirus Disease 2019 (COVID-19). *Centers for disease control and prevention.* https://www.cdc.gov/coronavirus/2019-ncov/prevent-getting-sick/diy-cloth-face-coverings.html (2020).

[4] Dhamecha TI, Singh R, Vatsa M, Kumar A (2014). Recognizing disguised faces: Human and machine evaluation. PLoS ONE.

[5] Stephan BCM, Caine D (2007). What is in a view? The role of featural information in the recognition of unfamiliar faces across viewpoint transformation. Perception.

[6] Kret M, De Gelder B (2012). Islamic headdress influences how emotion is recognized from the eyes. Front. Psychol..

[7] Carragher, D. & Hancock, P. J. Surgical face masks impair human face matching performance for familiar and unfamiliar faces (2020). 10.31234/osf.io/n9mt5. 10.1186/s41235-020-00258-x PMC7673975 33210257

[8] Gosselin F, Schyns PG (2001). Bubbles: A technique to reveal the use of information in recognition tasks. Vis. Res..

[9] Farah MJ, Wilson KD, Drain M, Tanaka JN (1998). What is ‘special’ about face perception?. Psychol. Rev..

[10] Maurer D, Grand RL, Mondloch CJ (2002). The many faces of configural processing. Trends Cogn. Sci..

[11] Wang R, Li J, Fang H, Tian M, Liu J (2012). Individual differences in holistic processing predict face recognition ability. Psychol. Sci..

[12] Busigny T, Joubert S, Felician O, Ceccaldi M, Rossion B (2010). Holistic perception of the individual face is specific and necessary: Evidence from an extensive case study of acquired prosopagnosia. Neuropsychologia.

[13] Ramon M, Busigny T, Rossion B (2010). Impaired holistic processing of unfamiliar individual faces in acquired prosopagnosia. Neuropsychologia.

[14] Ramon M, Rossion B (2010). Impaired processing of relative distances between features and of the eye region in acquired prosopagnosia—Two sides of the same holistic coin?. Cortex.

[15] Avidan G, Tanzer M, Behrmann M (2011). Impaired holistic processing in congenital prosopagnosia. Neuropsychologia.

[16] Tanzer M, Freud E, Ganel T, Avidan G (2013). General holistic impairment in congenital prosopagnosia: Evidence from Garner’s speeded-classification task. Cogn. Neuropsychol..

[17] Yin RK (1969). Looking at upside-down faces. J. Exp. Psychol..

[18] Farah MJ, Tanaka JW, Drain HM (1995). What causes the face inversion effect. J. Exp. Psychol. Hum. Percept. Perform..

[19] Freire A, Lee K, Symons LA (2000). The face-inversion effect as a deficit in the encoding of configural information: Direct evidence. Perception.

[20] Tanaka JW, Kaiser MD, Hagen S, Pierce LJ (2014). Losing face: Impaired discrimination of featural and configural information in the mouth region of an inverted face. Atten. Percept. Psychophys..

[21] Tanaka JW, Farah MJ (1993). Parts and wholes in face recognition. Q. J. Exp. Psychol..

[22] Duchaine B, Nakayama K (2006). The Cambridge Face Memory Test: Results for neurologically intact individuals and an investigation of its validity using inverted face stimuli and prosopagnosic participants. Neuropsychologia.

[23] Russell R, Duchaine B, Nakayama K (2009). Super-recognizers: People with extraordinary face recognition ability. Psychon. Bull. Rev..

[24] Bobak AK, Pampoulov P, Bate S (2016). Detecting superior face recognition skills in a large sample of young British adults. Front. Psychol..

[25] Murray E, Bate S (2020). Diagnosing developmental prosopagnosia: Repeat assessment using the Cambridge Face Memory Test. R. Soc. Open Sci..

[26] Corrow SL, Albonico A, Barton JJ (2018). Diagnosing prosopagnosia: The utility of visual noise in the Cambridge Face Recognition Test. Perception.

[27] JASP team. *JASP*. (2020).

[28] Herlitz A, Lovén J (2013). Sex differences and the own-gender bias in face recognition: A meta-analytic review. Vis. Cogn..

[29] Arizpe JM, Noles DL, Tsao JW, Chan AW-Y (2019). Eye movement dynamics differ between encoding and recognition of faces. Vision.

[30] Hsiao JH, Cottrell G (2008). Two fixations suffice in face recognition. Psychol. Sci..

[31] Shah P, Gaule A, Sowden S, Bird G, Cook R (2015). The 20-item prosopagnosia index (PI20): A self-report instrument for identifying developmental prosopagnosia. R. Soc. Open Sci..

[32] Shah P, Sowden S, Gaule A, Catmur C, Bird G (2015). The 20 item prosopagnosia index (PI20): Relationship with the Glasgow face-matching test. R. Soc. Open Sci..

[33] Ganel T, Goshen-Gottstein Y (2002). Perceptual integrality of sex and identity of faces: Further evidence for the single-route hypothesis. J. Exp. Psychol. Hum. Percept. Perform..

[34] Pomerantz JR, Garner WR (1973). Stimules configuration in selective attention tasks. Percept. Psychophys..

[35] Rossion B (2009). Distinguishing the cause and consequence of face inversion: The perceptual field hypothesis. Acta Psychol. (Amst.).

[36] Meissner CA, Brigham JC (2001). Thirty years of investigating the own-race bias in memory for faces: A meta-analytic review. Psychol. Public Policy Law.

[37] Michel C, Rossion B, Han J, Chung C-S, Caldara R (2006). Holistic processing is finely tuned for faces of one’s own race. Psychol. Sci..

[38] Young AW, Hellawell D, Hay DC (1987). Configurational information in face perception. Perception.

[39] Behrmann M, Avidan G (2005). Congenital prosopagnosia: Face-blind from birth. Trends Cogn. Sci..

[40] Duchaine BC, Nakayama K (2006). Developmental prosopagnosia: A window to content-specific face processing. Curr. Opin. Neurobiol..

[41] McBain R, Norton D, Chen Y (2009). Females excel at basic face perception. Acta Psychol. (Amst.).

[42] Arizpe JM, Walsh V, Yovel G, Baker CI (2017). The categories, frequencies, and stability of idiosyncratic eye-movement patterns to faces. Vis. Res..

[43] Lane J (2018). Impacts of impaired face perception on social interactions and quality of life in age-related macular degeneration: A qualitative study and new community resources. PLoS ONE.

[44] Ma DS, Correll J, Wittenbrink B (2015). The Chicago face database: A free stimulus set of faces and norming data. Behav. Res. Methods.

