# Supplementary material for: The COVID-19 pandemic masks the way people perceive faces
Source: Sci Rep. 2020 Dec 21;10:22344. doi: 10.1038/s41598-020-78986-9 (PMC7752904; doi:10.1038/s41598-020-78986-9)
Supplement: Supplementary file 1 — Supplementary Information. [file 41598_2020_78986_MOESM1_ESM.pdf]

Supplementary Materials - The COVID-19 pandemic masks the way people perceive faces

Erez Freud, Andreja Stajduhar, R. Shayna Rosenbaum, Galia Avidan and Tzvi Ganel

Repeated Measures ANOVA – Experiment 1

Within Subjects Effects

	Sum of Squares	df	Mean Square	F	p	η^2_p
Orientation	17272.539	1	17272.539	629.555	< .001	0.685
Orientation ∗ group	1624.817	1	1624.817	59.222	< .001	0.170
Orientation ∗ gender	95.187	1	95.187	3.469	0.064	0.012
Orientation ∗ group ∗ gender	0.999	1	0.999	0.036	0.849	0.000
Residual	7929.031	289	27.436			

Note. Type III Sum of Squares

Between Subjects Effects

	Sum of Squares	df	Mean Square	F	p	η^2_p
group	4677.136	1	4677.136	46.686	< .001	0.139
gender	3257.044	1	3257.044	32.511	< .001	0.101
group ∗ gender	69.422	1	69.422	0.693	0.406	0.002
Residual	28952.914	289	100.183			

Note. Type III Sum of Squares

Repeated Measures ANOVA – Experiment 1 – analysis by CFMT level

Within Subjects Effects

	Sum of Squares	df	Mean Square	F	p	η^2_p
Orientation	8.440	1	8.440	621.461	< .001	0.681
Orientation ✻ group	0.770	1	0.770	56.668	< .001	0.163
Residual	3.952	291	0.014			
CFMT phase	76.829 [a]	3 [a]	25.610 [a]	1693.002 [a]	< .001 [a]	0.853
CFMT phase ✻ group	0.617 [a]	3 [a]	0.206 [a]	13.592 [a]	< .001 [a]	0.045
Residual	13.206	873	0.015			
Orientation ✻ CFMT phase	1.778 [a]	3 [a]	0.593 [a]	58.903 [a]	< .001 [a]	0.168
Orientation ✻ CFMT phase ✻ group	0.373 [a]	3 [a]	0.124 [a]	12.348 [a]	< .001 [a]	0.041
Residual	8.784	873	0.010			

Note. Type III Sum of Squares

[a] Mauchly's test of sphericity indicates that the assumption of sphericity is violated ($p < .05$).

Between Subjects Effects

	Sum of Squares	df	Mean Square	F	p	η^2_p
group	1.976	1	1.976	34.196	< .001	0.105
Residual	16.816	291	0.058			

Note. Type III Sum of Squares

Difference between groups per CFMT level

Simple Main Effects - group

Level of CFMT phase	Sum of Squares	df	Mean Square	F	p
1	0.390	1	0.390	13.049	< .001
2	1.567	1	1.567	50.970	< .001
3	0.612	1	0.612	19.727	< .001
4	0.023	1	0.023	2.025	0.156

Note. Type III Sum of Squares

Repeated Measures ANOVA across experiments 1 and 2

Within Subjects Effects

	Sum of Squares	df	Mean Square	F	p	η^2_p
Orientation	20487.906	1	20487.906	692.228	< .001	0.587
Orientation ✻ group	2265.301	3	755.100	25.513	< .001	0.136
Orientation ✻ gender	54.257	1	54.257	1.833	0.176	0.004
Orientation ✻ group ✻ gender	158.431	3	52.810	1.784	0.149	0.011
Residual	14443.353	488	29.597			

Note. Type III Sum of Squares

Between Subjects Effects

	Sum of Squares	df	Mean Square	F	p	η^2_p
group	6000.554	3	2000.185	22.294	< .001	0.121
gender	2837.666	1	2837.666	31.629	< .001	0.061
group ✻ gender	867.335	3	289.112	3.222	0.022	0.019
Residual	43782.145	488	89.718			

Note. Type III Sum of Squares

Inversion effect per group:

Simple Main Effects - Orientation

Level of group	Sum of Squares	df	Mean Square	F	p
mask	4134.321	1	4134.321	163.812	< .001
mask study	2764.711	1	2764.711	70.514	< .001
mask test	3143.076	1	3143.076	119.977	< .001
no mask	14806.254	1	14806.254	499.893	< .001

Note. Type III Sum of Squares
